# Editorial: Community series in insights of gut microbiota: probiotics and bioactive compounds, volume II

**DOI:** 10.3389/fmicb.2023.1188151

**Published:** 2023-06-13

**Authors:** Smith Etareri Evivie, Shyamchand Mayengbam

**Affiliations:** ^1^Department of Food Science and Technology, University of Benin, Benin City, Nigeria; ^2^Department of Animal Science, University of Benin, Benin City, Nigeria; ^3^Key Laboratory of Dairy Science, Northeast Agricultural University, Harbin, China; ^4^Department of Biochemistry, Memorial University of Newfoundland, St John's, NL, Canada

**Keywords:** probiotics, gut microbiota, research, innovation, therapy

The last two decades have seen a surge of investigations into the efficacy of probiotics and bioactive compounds, which have shown potential for industrial and therapeutic applications. Probiotics are a type of live bacteria that are beneficial to human health. They have been demonstrated to improve immune function and reduce inflammation through the production of various pathogen-inhibiting compounds, including bacteriocins, organic acids, and exopolysaccharides (Zhou et al., 2022). Recently, probiotics supplementation have gained significant interest for their potential in maintaining human health and treating diseases such as inflammatory bowl disease, colitis, non-alcoholic steatohepatitis, and rheumatoid arthritis. This series covers seven original research and one review articles investigating diverse probiotics, their potential mechanism of actions as well as different approaches of analyzing microbial diversity.

The role of probiotics, specifically the gut microbiome, in modulating host immune and inflammatory function, is continually being discovered. Probiotics can improve gut integrity by increasing the production of mucins and tight junction proteins, which are essential for maintaining intestinal barrier function (Gou et al., 2022). They have also been reported to modulate the immune system by activating various signaling pathways, including the maturation of B cells into immunoglobulin (Ig) and thus increase the production of Ig (Mazziotta et al., 2023). Zhao et al. explored the *in vivo* regulatory effects of a polysaccharides extracted from an edible fungus, *Auricularia cornea var Li*., on host immunity and gut microbiomes in a rodent model of immunosuppression. The polysaccharide increases the serum levels of immunoglobulins (Ig) such as (IgA), IgG and IgM.

Some strains of probiotics have been found to reduce pro-inflammatory cytokines such as TNF-α and IL-6, while others have been shown to increase anti-inflammatory cytokines such as IL-10 (Mazziotta et al., 2023). Interestingly, Ma et al. have also conducted a study on the immunomodulatory effects of a combination of complex probiotics consisting of *Bifidobacterium animalis* subsp. *lactis* XLTG11, *Lacticaseibacillus casei*, and *Lactiplantibacillus* using an immunosuppressed mice model. This combination normalized the blood cytokine levels, restored gut microbiota structure, and increase the relative abundances of short-chain fatty acid (SCFA)-producing bacteria. SCFAs are known to be important in maintaining gut health and have been linked to several beneficial effects, including reducing inflammation. These mechanisms indicate that a multi-strain probiotic might be more effective than individual strains in modulating immune systems.

One of the most challenging chronic intestinal diseases is the ulcerative colitis (UC), which is associated with imbalance in immune responses and mucosal barrier damage. There is a growing research opinion that probiotic bacteria can be a potential alternative therapy for UC. Wang et al. identified the mechanistic role of *Lactiplantibacillus plantarum* L15 using a mouse model of colitis. Their findings revealed that this probiotic bacterial strain could substantially decrease infection parameters like myeloperoxidase activity, disease activity index, pro-inflammatory cytokine (TNF-α, IL-1β, and IL-6) levels while reducing the transfer of NF-κB p65 to the nucleus. Additionally, this strain increased colon length, improved the gut microbiota composition, and increased SCFAs content in the colon.

The beneficial roles of probiotics have also been extensively studied in some of the chronic liver diseases such as non-alcoholic fatty liver disease. It is a spectrum of diseases that encompasses a range of conditions including non-alcoholic steatohepatitis (NASH). NASH is characterized by liver inflammation caused by excessive fat deposition (Pafili and Roden, 2021), and it remains a global health burden due to the lack of approved medications for its treatment. Dysbiosis of gut microbiota can have multiple negative impacts on the liver function and plays key role in the pathophysiology of NASH (Fang et al., 2022). Yang et al. tested the effects of a probiotic yeast, *Saccharomyces boulardii*, on a C57/BL6 mouse model of diet-induced NASH. They found that supplementing with the yeast resulted in reversal of liver function, decreased inflammation with less hepatic steatosis, attenuate gut leakage, and thus protected the mice against NASH. Obesity is one of the major causes of NASH. Several clinical studies have reported that a high-fat diet (HFD) may be responsible for obesity-linked impairment in fat metabolism. Kim et al. found that HFD increases cholesterol accumulation in the body and causes inflammation of gut mucosal cells. Although certain probiotic such as *Bifidobacterium longum* was helpful in lowering cholesterol and inflammation, probiotic-mediated actions on these pathways as potential anti-obesity effects are yet to be fully described.

Could probiotics interacting with a complex mix of intestinal microbes be a potential therapy for rheumatoid arthritis (RA)? The link between gut microbiome dysbiosis and the onset of RA is an area of intense research. Emerging evidence suggests that probiotics could modulate the gut microbiota and the immune system, potentially improving RA outcomes (Bungau et al., 2021). Opoku et al. has comprehensively reviewed the roles of the gut dysbiosis with the initiation and progression of RA and how probiotic supplementation can attenuate it. RA is a systemic autoimmune disease and with the long-term complications and progressive disability, it can be another social-economic burden. The authors showed that probiotics could ameliorate symptoms of RA by correcting gut dysbiosis, modulating immune system, and reducing inflammatory cytokines. It is noted that these findings are promising, but strain-specific roles of these probiotics warrant further investigations.

The composition of microbiome is unique in each individual and microbiome could play a significant role in shaping their metabolic phenotype. Shaping of microbial composition starts from the early stage of our life (Gilbert et al., 2018). The human breast milk microbiome (BMM) can help in seeding and nurture the gastrointestinal colonization of children. Quantitative measurement of population-level diversity can be quite different from individual-level diversity analysis. Diversity analysis of BMM across individual in a population or cohort requires special analytical approaches. Chen et al. reanalyzed a dozen of dataset of 2,115 human BMM samples with diversity-area relationship (DAR) to explore the spatial scale of species diversity. They found that inter-individual heterogeneity in diversity was constant, but the population potential diversity was different among 30% of the pairwise comparison. Their data also provide relationship between the stability of BMM and host health. Lastly, arguing that the shared species analysis (SSA) computational method may be more holistic than the standard de facto gut microbiome analysis protocols, Ma investigated the effects of ethnicity and lifestyles on the structure of gut microbiomes by reanalyzing the datasets of a large Chinese cohort with 300+ individuals covering seven biggest ethnic groups (>95% Chinese population). The findings indicate that the SSA was more effective than the current diversity analysis protocols in providing more information on ethnicities and lifestyle-based bacterial community structures.

Overall, this Research Topic has presented a comprehensive collection of data on probiotics and the human microbiome. While some findings may appear to challenge our current understanding, it is important for us, as scientists, to approach them with an open mind and be motivated to conduct more detailed studies. The beneficial roles of probiotics and the human microbiome have been well-documented. Additionally, there have been recommendations for novel approaches to analyzing microbial diversity for future research; however, these approaches need to be validated.

## Author contributions

All authors listed have made a substantial, direct, and intellectual contribution to the work and approved it for publication.
